# DNA Methylation Patterns Between Serous Uterine and Ovarian Carcinomas Indicate Shared Markers with Fallopian Tube Epithelium and Distinctions Consistent with Histopathology

**DOI:** 10.3390/genes17101160

**Published:** 2026-09-22

**Authors:** Emily Kistner-Griffin, Mirna Mina-Abouda, Lulu Serang, Rachel Choe, Wendy Y. Zhang, David P. Mysona, Joe R. Delaney

**Affiliations:** 1 Department of Public Health Sciences, Medical University of South Carolina, Charleston, SC 29425, USA; 2 Hollings Cancer Center, Medical University of South Carolina, Charleston, SC 29425, USA; 3 Department of Biochemistry and Molecular Biology, Medical University of South Carolina, Charleston, SC 29425, USA; 4 Division of Gynecologic Oncology, Obstetrics and Gynecology, Medical University of South Carolina, Charleston, SC 29425, USA

**Keywords:** uterine serous carcinoma, high-grade serous ovarian cancer, DNA methylation, epigenetics, cell-of-origin

## Abstract

Background/Objectives: Uterine serous carcinoma (USC) is a deadly form of Type II endometrial cancer of rising incidence in the USA. It is currently thought to originate in the uterus, but emerging evidence suggests USC etiology is more complex and may involve the fallopian tube. DNA methylation is an inheritable suppressive epigenetic marker that can differentiate cellular origins, so we opted to perform a study comparing DNA methylation of USC to the fallopian tube and uterus. Methods: DNA methylation profiles were compared between USC, fallopian tube epithelium (FTE), normal uterus, and a cancer known to primarily originate from FTE: high-grade serous ovarian cancer (HGSC). Hierarchical clustering of DNA methylation was performed. Variably DNA-methylated genes were defined for tumors and the FTE relative to normal uterus. Results: Hierarchical clustering of USC separated USC from normal uterus but showed similarity with some FTE profiles, such as HGSC. Differentially methylated genes in USC correspond to both known and novel genes related to USC biology. Known biomarkers included hypomethylation of *PAX8* in both HGSC and USC, which is often used in histopathology. Another known histopathological marker is WT1, which is more often expressed in HGSC than USC. By DNA methylation, hypomethylation was observed with *WT1* in HGSC, whereas in USC WT1 hypermethylation was observed. Other novel markers of interest are discussed within. Conclusions: There is substantial overlap in DNA methylation biomarkers between the fallopian tube epithelium and USC tumors, just like the known FTE-originating carcinoma: HGSC. These results are supportive of the possibility of certain USCs arising first from the FTE (thereby retaining inheritable DNA methylation markers from the FTE) and then metastasizing to the uterus.

## 1. Introduction

While most cancers are decreasing in incidence in the US, a few outliers require additional attention due to their unusual growth in incidence. One of those is Type II endometrial cancer, particularly of the papillary serous, or simply serous, subtype. Uterine serous carcinoma (USC) is a deadly form of endometrial cancer: often referred to as 40% of deaths despite being only 10% of cases [1,2]. However, even this grave calculus is changing for the worse: from 2001 to 2021, type II endometrial cancer (largely USC) increased from 34 per 1,000,000 women to 63 per 1,000,000 women, an 85% increase [3]. In 2025 endometrial cancer moved from the sixth most deadly cancer in women to the fifth most deadly. Understanding why incidence is increasing is multifactorial and interdisciplinary. One particularly important question to address is where USC originates from, under the lens of recent molecular technologies and associated data.

A growing body of evidence suggests the origin of uterine serous carcinoma may not always be the endometrium itself. Retrospective studies have revealed a link between tubal ligation, which may prevent intraepithelial metastasis from the fallopian tube, and reduced rates of endometrial cancer [4]. In a carefully controlled cohort study of tubal ligation, the hazard ratio for Type I endometrioid uterine cancer was 0.78, but the hazard ratio was strikingly 0.25 for Type II, typically p53-mutant serous, uterine cancer [5], demonstrating dramatically reduced USC incidence after tubal ligation. Our laboratory published a mouse model of spontaneous cancer derived from the *Ovgp1* locus, which predominantly expresses on the fallopian tube epithelium, and found high rates of cancer cell clusters intercalated into the uterine epithelium [6]. Upon dissection and growth of epithelial cancer cells in the laboratory, the uterine cancer cells had a remarkably similar transcriptional pattern to human high-grade serous ovarian carcinomas and uterine serous carcinomas alike.

Currently, USC is thought to arise in the endometrium, and HGSC is thought to arise primarily in the fallopian tube epithelium (FTE). However, given the above-mentioned studies, it is possible that USC tumors first arise in the fallopian epithelium and metastasize to the uterus. USC and HGSC share many characteristics with only a few defining differences, as both diseases are heterogeneous in biomarker expression [7]. USC [2,8,9] and HGSC [10,11,12] are both marked by nuclear atypia, ubiquitous p53 mutations and aberrant immunostaining, papillary and solid architecture, and extensive peritoneal metastases. USC [13] and HGSC [14] are treated similarly. Both are surgically resected with a total hysterectomy, bilateral salpingo-oophorectomy (BSO), and cytoreductive surgery, followed by adjuvant platinum–taxane doublet chemotherapy. Interestingly, these cancers are also difficult to discriminate pathologically, with pathologists evaluating the endometrium for serous endometrial intraepithelial carcinoma (SEIC) lesions in the uterine surface epithelium to potentially indicate USC or for serous tubal intraepithelial carcinomas (STIC) in fallopian fimbriae to indicate HGSC [15,16]. However, SEIC and STIC can coexist [17,18]. Intraluminal tubal and endometrial floating tumor fragments have been observed, which could originate from either the endometrium or fallopian tube and remain controversial regarding their origin [8,19]. Immunohistochemically, WT1 is most often present in HGSC and most often absent in USC [20]; however, there are cases wherein WT1 stains positively in USC [17] and cases wherein WT1 staining is absent in HGSC [21]. Molecular studies better delineating these diseases may help better decipher treatment targets which are shared or unique to individual disease sites.

A prediction from the model that uterine serous carcinomas are, at times, derived from the fallopian tube epithelium is that inheritable epigenetic markers in these USC tumors are also found in the normal fallopian tube. Another prediction is that USC epigenetic markers are more similar to the fallopian tube than to the uterus. If both HGSC and USC are derived from the FTE, then both types of cancer should share epigenetic markers derived from the FTE. There are many epigenetic markers that are inherited following cell division. One such marker with ample data available to study is CpG methylation. Here, a cytosine in DNA is methylated by a DNA methyltransferase (DNMT) on the fifth carbon of the cytosine ring, creating 5-methylated cytosine (5-Me-C or 5-mC), which occurs most often in CG-rich sequences termed CpG islands [22]. DNA methylation helped better classify ovarian cancers, particularly those with original cancer of unknown primary (CUP) status, noting *PAX8* DNA methylation as a fimbrial-origin HGSC biomarker [23]. TCGA analysis publications previously noted that the serous subtype of endometrial cancers clustered separately from other endometrial cancers [24] and was more ‘normal-like’ [25]; i.e., had less hypermethylation than other subtypes. The less hypermethylated phenotype of USC was identified previously in an endometrial-specific analysis of an independent cohort [26]. Previous to our study here, a lack of FTE origin DNA methylation profiles limited a study examining a comparison of uterine origin versus fallopian origin for USC.

Here, we make the directed comparison of USC to two possible origin sites: FTE or uterus. We find that the DNA methylation patterns of USC tumors more closely resemble FTE than the uterus by hierarchical clustering and shared gene methylation status. We additionally find clear differences between USC and HGSC, which explain some of the histopathological findings that currently discriminate the diseases. Overall, the possibility of USC originating from the FTE remains supported by DNA methylation data.

## 2. Materials and Methods

Fallopian tube tissue DNA methylation profiles were downloaded from the Gene Expression Omnibus (GEO) under accession numbers GSE65820 (n = 6) and GSE81228 (n = 5). Uterine normal tissue (n = 47), p53-mutant uterine serous carcinoma (USC) tumor samples (n = 37), and high-grade serous ovarian carcinoma (HGSC) tumor samples (n = 611) were obtained from The Cancer Genome Atlas (TCGA) via the NCI Genomic Data Commons (GDC). All DNA methylation data were generated using the Illumina Human Methylation 450k array. Raw IDAT files for the GEO datasets were processed using the minfi package in R, with background correction and control normalization [27]. TCGA methylation data were accessed as pre-processed beta values. Datasets are reported with hg19 coordinates.

To ensure balanced group representation in downstream visualization, a random subset of 63 HGSC tumor samples was selected from the full TCGA ovarian dataset, approximately matching the size of the other tissue groups. Due to variable probe coverage across datasets, analysis was restricted to probes with complete data across all samples. Missingness differed substantially by dataset: GEO-derived fallopian tube samples (GSE65820, GSE81228) had negligible missingness (0.007% and 0.006% respectively), uterine normal TCGA samples had 16.2% average per-probe missingness, and UCEC p53 tumor samples had 46.1% average per-probe missingness distributed across different probes in different samples. Ovarian HGSC tumor samples showed the highest missingness at 93.7%, which could reflect lower DNA quality in this tumor type. Because missingness was high and distributed across the majority of probes in the TCGA datasets, probes with any missing data were excluded, retaining only probes with complete data across all samples and datasets. After requiring complete data across all samples, 19,733 probes were retained for analysis of ovarian HGSC tumor samples and 21,476 probes were retained for analysis of UCEC p53 tumor samples.

Prior to clustering, technical batch effects arising from differences in dataset origin were corrected using the ComBat function, an empirical Bayes method implemented in the sva R package (version 4.5.2) [28]. Dataset source was used as the batch variable, with GSE65820 and GSE81228 samples assigned to a GEO batch and TCGA samples assigned to a separate batch. To visualize the impact of this batch correction, we generated Supplemental Figure S1, containing hierarchical clustering before batch correction and principal component analysis (PCA) plots before and after batch correction. The 1000 probes with the highest variation across all samples were used for unsupervised hierarchical clustering and visualization. Clustering was performed using Ward’s minimum variance method (Ward.D2) with Euclidean distance. Heatmap colors represent row-scaled methylation beta values (z-scores), where red indicates higher methylation relative to the mean across all samples for a given probe, and blue indicates lower methylation relative to the mean. All analyses were conducted using R version 4.5.2.

To enable rapid clustering analyses on the presented data and others by wet lab scientists, bioinformaticians, and statisticians, we release an R Shiny App to perform hierarchical clustering. It is made available for free use at https://www.delaneyapps.com/ and all associated application code is available on https://github.com/jrdelaney/Published_Code/tree/main/Hierarchical_Clustering_ShinyApp (access date: 1 September 2026). The clustering in Supplemental Figure S2 was generated through this associated code.

To evaluate differential methylation within the top 1000 most variable CpGs, two-sample *t*-tests were performed comparing mean beta values between groups of interest. Specifically, GEO-derived fallopian tube samples and HGSC tumor samples were compared to uterine normal samples, and the same approach was applied when comparing GEO fallopian tube and UCEC p53 tumor samples to uterine normal samples. FDR correction was performed using the Benjamini–Hochberg method, with FDR ≤ 0.05 considered significant. To explore concordance between the two analyses, mean beta value differences were compared by estimating the Pearson correlation coefficients among the subset of CpGs that reached FDR ≤ 0.05 in both comparisons.

## 3. Results

### 3.1. DNA Methylation Datasets for FTE, USC, and HGSC

Since the central hypothesis we want to test is that USC may come from the FTE and therefore retains some biomarkers of the FTE, we sought sources of DNA methylation (5-methylcytosine; 5-mC) data that were truly FTE specific rather than paired normals to HGSC, which are often ovaries rather than fallopian tubes. Few datasets existed, but fortunately two were usable in our analysis: GSE81228 and GSE65820. In GSE65820, fallopian tube epithelial cells (samples AOCS173-178) were processed for methylated DNA and analyzed on Infinium Human Methylation 450K BeadChips [29]. In GSE81228, fallopian tube epithelial cells were independently collected and processed similarly from flash-frozen tissue [30]. For normal uterus, ample normal uterine samples were available from the primary endometrial cancer dataset.

For tumor DNA methylome data, we utilized TCGA datasets. The HGSC dataset [31] was used, and 63 samples were randomly sampled, so it was closer in size to the comparison dataset: FTE (n = 11 samples) and normal uterus (n = 47). Different random sampling produced similar results (Supplemental Figure S1C). Our USC dataset consisted of the UCEC TCGA dataset for samples that were (i) indicated as serous in clinical data and (ii) had a mutation in p53 (n = 37 samples).

### 3.2. Hierarchical Clustering of HGSC with Normal FTE and Normal Uterus Reveals Separate Upregulated and Downregulated DNA Methylation Biomarkers of HGSC

HGSC is known to originate in many cases from FTE, which allows for conserved, inherited DNA methylation patterning. However, some changes are likely to occur during tumor development. To discover such biomarkers that are (i) shared between FTE and HGSC, but different in the uterus or (ii) distinct in only HGSC and not shared with normal FTE or uterus, samples were compared using hierarchical clustering with the top 1000 most variable DNA methylation markers (see Materials and Methods) (Figure 1). Some FTE samples clustered well within the HGSC group, as expected from HGSC’s FTE origin. However, since this clustering analysis utilized discriminatory markers, not all FTE clustered within the HGSC group. This is also expected, as some DNA methylation biomarkers of HGSC should differ from normal tissues. HGSC exhibited both hypermethylated and hypomethylated regions, consistent with coincident epigenetic suppression of tumor suppressors and de-repression of oncogenes [32]. The top 1000 most variable DNA methylation markers used in this analysis create a useful set of probes that can be informative for both (i) FTE-HGSC shared and (ii) HGSC unique biomarkers.

### 3.3. Hierarchical Clustering of USC with Normal FTE and Normal Uterus Results in FTE-Proximal Clustering of Tumors

USC is currently thought to originate from the uterus, so it would be surprising if DNA methylation patterning was more similar to FTE than uterus. That is, unless USC originates from the FTE, in which case normal FTE would exhibit DNA methylation patterns more similar to USC than normal uterus. To test these two possibilities, a similar top 1000 variable biomarker approach was used, comparing USC to normal FTE and uterus. Surprisingly, hierarchical clustering of these markers placed USC closer to normal FTE samples than to normal uterus samples (Figure 2). This clustering is consistent with the FTE origin of USC hypothesis. The same four FTE samples clustered within USC tumors as those that clustered within HGSC tumors, both in the hierarchical clustering and by PCA (Supplemental Figure S1). Also consistent with tumor-specific DNA methylation patterns, the clustering showed clearly less hypomethylation in some FTE samples than USC samples. *PIK3CA* mutation status, a known distinct oncogenic driver mutated often in USC but infrequently in HGSC [6], is plotted for reference, but did not drive clustering of 5-mC patterns. Thus, like HGSC, some DNA methylation biomarkers were (i) shared between the FTE and USC, whereas others were (ii) unique to USC relative to both normal FTE and normal uterus. Comparing genes with these specific variably methylated regions is of interest to determine if such patterns are relevant to tumor development.

### 3.4. Identification of Shared USC-HGSC DNA Methylation Biomarkers Present in FTE

If USC and HGSC share an FTE origin, then there should be specific DNA methylation biomarkers that are different from normal uterus in both analysis datasets. The most pertinent would be those biomarkers associated with specific genes. Hundreds of genes were dysregulated by DNA methylation in HGSC (Figure 3A) and USC (Figure 3B) relative to normal uterus and similarly dysregulated in FTE. Sure enough, 92 genes fit these criteria for upregulated DNA methylation (typically associated with downregulated gene expression). The overlap in genes significant in the same direction was substantial compared to a randomly picked gene dataset from the genes with overlap between the two datasets (same gene, same significance and direction: 341 (91.7%), not same significance and direction: n = 31 (8.3%); random pick same = 182, random pick not same = 190; *p* < 1.12 × 10^−39^ by Fisher’s exact test, using Supplementary Tables S1–S3 data). GOrilla pathway analysis [33] did not identify significant shared hypomethylated gene sets, but shared nominally significant hypermethylated gene sets included protein–DNA complex assembly (*p* < 7.3 × 10^−5^; FDR < 0.33), chromatin silencing (*p* < 3.3 × 10^−4^; FDR < 0.25) and regulation of transcription, DNA-templated (*p* < 4.4 × 10^−4^; FDR < 0.22). Specific genes were perhaps not the most immediately obvious biomarkers. HGSC is known to be driven by activation of *MYC*, *CCNE1*, and *PTK2*, and by suppression of *NF1*, *BRCA1/2*, *RB1*, and *TP53*, among other drivers [31]. However, these are not likely suppressed or de-repressed often by 5-mC changes, as none were identified as significantly different from the normal uterus within HGSC tumors within the variably methylated set. Thus, the USC/HGSC/FTE shared genes found here may be more developmentally regulated, as many markers are shared with normal FTE DNA methylation profiles.

Hypomethylation of the top 1000 variable DNA methylation markers was more consistent between USC and HGSC than hypermethylation (Figure 4). Impactful genes are amongst these changes, including *VHL* (a tumor suppressor involved in hypoxia response), *GNAS* (a tumor suppressor related to PKA and Sonic hedgehog signaling), *TMEM139* (a tumor suppressor acting to suppress epithelial-to-mesenchymal transition (EMT)), amongst others. Interestingly, immunomodulatory factors are within this group, including *SEMA4A*, *SERPINB5*, and *CD300E*. RAB25 is a ciliated epithelial cell marker, including uterine ciliated cells [34], important for endocytosis and oncogenic signaling through anti-apoptotic and ERK signaling [35], and was observed as hypomethylated in both tumor types. *CLDN16* was an interesting shared de-repressed gene by DNA hypomethylation. It is a cell polarity factor [36], overexpressed in ovarian cancers with PI3K signaling [37], and more highly expressed in recurrent tumors [38]. *CLDN16* has not been widely reported in the endometrial cancer literature and may be a USC biomarker related to HGSC-like biology. *AGR2*, significantly de-repressed by DNA methylation in USC, HGSC, and FTE compared to normal uterus, is an endoplasmic reticulum resident protein involved in mucin maturation and export for mucinous processes [39]. It is necessary for proliferation in a variety of adenocarcinomas [40] and was found as a therapeutic target using clear cell ovarian cancer organoids [41]. While the concordance of HGSC and USC hypomethylation is consistent with a shared origin, the loss of beta-value correlation with hypermethylation may indicate that if both tumors arise from the FTE, hypermethylation processes may be involved during the migration of cells into the uterus to form the primary tumor.

Shared hypermethylated genes also appear relevant for the shared USC and HGSC biology. *SLC22A18* was the most hypermethylated gene relative to normal uterus in HGSC and the second most hypermethylated gene in USC. *SLC22A18* transporter suppression was directly shown to induce proliferation, migration, and invasion of colon cancer cells [42], which notably also originate from polyps. The top USC hypermethylation biomarker (second most hypermethylated in HGSC), *ZNF154*, has been observed as methylated in serum and tumor for ovarian and other solid tumor cancer patients [43], and directly demethylating *ZNF154* in cell culture allowed for ROMO1 repression and reduced ERK signaling [44]. A published poster abstract noted that serum *CDO1* and *HOXA9* hypermethylation can potentially improve early diagnosis of ovarian cancer [45], but it was also found in the USC dataset as hypermethylated. *GATA4*, a critical embryonic patterning transcription factor, is lost in serous ovarian cancers [46] and was also found here as hypermethylated in USC. RAP1 signaling, which can be counteracted by HGSC/USC hypermethylated tumor suppressor RAP1GAP, is known to contribute to oncogenic signaling in ovarian cancer [47]. XAF1, another dual HGSC/USC hypermethylated tumor suppressor, acts as an anti-apoptotic protein through XIAP, particularly in ovarian cancer progression [48]. Thus, shared hypermethylated genes between HGSC and USC are both cancer-wide regulatory genes and genes that have a published association with ovarian cancer biology.

### 3.5. Identification of Discriminating USC and HGSC DNA Methylation Biomarkers

While PAX8 is a marker shared amongst gynecologic malignancies, WT1 is used in immunohistochemical staining strategies to discriminate some ovarian tumors from endometrial tumors. Endometrioid ovarian cancers most often do not express WT1, whereas serous ovarian cancers do express WT1 [49]. Between serous endometrial cancer and serous ovarian cancer, one study reports > 90% positive WT1 staining with serous ovarian cancer but only 20% positive WT1 staining in USC [20]. Figure 3B indicates WT1 is more often suppressed by DNA methylation (more positive methylation beta values). Interestingly, the WT1 DNA methylation marker that was most often hypermethylated in USC was located on an alternate exon 1 transcriptional start site for USC (Supplemental Table S1). Isoform-specific WT1 antibodies or transcript-based assays [50] may be able to discriminate such differential transcript expression effects in future studies. Taken together with previous studies, while WT1 may help identify some gynecologic cancer cases of unclear origin, it remains an imperfect marker.

For HGSC-unique DNA methylation markers, a select few are consistent with previous studies and are likely useful to discriminate between HGSC and USC. Publication-supported genes are summarized in Figure 5 and described here, while the full gene dataset is supplied as Supplemental Table S2. Mesothelin (*MSLN*) is often overexpressed in HGSC and plays a role in tumor progression [51], is hypomethylated only in HGSC. *CDCP1*, hypomethylated in HGSC only, expresses a cell surface protein expressed in many cancers, but also notably in HGSC, and was shown to be related to progression in HGSC patient-derived xenografts [52]. *GREB1*, hypomethylated in HGSC only, is expressed in ovarian cancers, including HGSC, and can heighten estrogen-dependent signaling for estrogen-dependent cancers or act independently of the estrogen receptor to activate proliferative and migratory signaling in HGSC [53]. *KLK6*, hypomethylated in HGSC only, is a serum-present biomarker studied previously in HGSC, which may improve detection panels [54].

Conversely, suppressed genes (hypermethylation) unique to HGSC map to tumor-suppressor functions. *FOXD3* was previously identified as hypermethylated in HGSC and subsequently tested as regulating apoptosis, proliferation, and migration [55]. *EPB41L3* has also been shown to be hypermethylated in HGSC and acts as a tumor suppressor in HGSC by increasing apoptosis in spheroid conditions [56]. *SOX1* has been placed in hypermethylation profiles for HGSC [57] and may act in tumor stemness through HES1 regulation [58]. ADAMTS8 has not been implicated in HGSC but has been shown to be involved in apoptotic signaling as well as antagonizing EGFR/MEK/ERK signaling [59]. *EI24* is similar in function but acts through NFκB [60]. *AKAP12* is also not well studied in HGSC, but acts to dilute out oncogenic proteins involved in HGSC, such as SRC, through scaffolding functions [61].

USC may be aided in detection and understanding of etiology through the uniquely hypomethylated genes. Novel biomarkers may include *DPP4*/DPPIV, which is a surface glycoprotein shown to enhance endometrial cancer proliferation, invasion, and tumorigenesis *in vivo* [62]. MMP26 was previously shown to have higher expression in endometrial cancer compared to normal endometrium [63]. *TINAG* was shown to be essential for PI3K/AKT signaling and resulting proliferation, invasion, and migration in hepatocellular carcinoma [64], but not yet in endometrial cancer. *ZFP42*/REX1 is poorly characterized in endometrial cancer but notably upregulated during endometriosis [65] and acts as a known stemness gene for embryonic stem cells [66] and an EMT promoter in cervical cancer [67]. These markers were all found to be hypomethylated in USC and FTE, but not HGSC.

USC-specific hypermethylated genes are equally interesting to inform tumor biology. *WT1* is a clear, known example, discussed above. Decreased *HOXD10* expression is associated with endometrial adenocarcinomas but not mucinous endometrial cancers [68], and is a known tumor suppressor in multiple solid tumors. *LEFTY2* in endometrial cancer inhibits FAK as a tumor suppressor [69]. LEFTY2 is also a cytokine released before menstrual bleeding [70], and thus, with further study, may rationally serve to discriminate USC from HGSC in complex cases. It should be noted that these analyses are based on variably methylated probes, which differ between cancer types, and are not comprehensive or specific tests of unique markers: follow-up assays with immunohistochemistry, for example, must be performed to validate the candidate markers analyzed here. For a table of all overlapping or USC/HGSC-unique differentially DNA methylated genes, please refer to Supplementary Table S3.

## 4. Discussion

While it took two decades to firmly establish a field consensus that most HGSC originate from the FTE, few are looking into the possibility of a non-uterine origin site of USC. Our results indicate the FTE, USC, and HGSC all share important DNA methylation biomarkers distinct from the uterus. On a molecular basis, a large proportion of shared DNA methylation biomarkers were genes that regulate ERK signaling. The data are consistent with USC etiology models in which USC originates from the FTE in at least some cases, but do not currently rule out either model of development.

Multiple models of cancer cell metastases through the gynecologic compartments are possible for HGSC and USC. Certain models are less likely to be explained by the data presented here. First, HGSC is now well-established to originate in most cases from the FTE, where it metastasizes to the ovary [71], and then to distal peritoneal sites, sometimes including the myometrium [20]. However, HGSC may not always follow this evolutionary tree in all patients; some mutational phylogenetic studies indicate the earliest mutations in fact are on the ovary, and STIC lesions form secondarily or in parallel [72]. USC is thought to originate most often in uterine polyps; however, given the DNA methylation data presented here, it is more likely that pre-cancerous lesions emerge from the FTE and migrate into the uterus, where they seed polyps and grow into uterine tumors. The migration may be through the floating intra-luminal cell clusters sometimes observed by histology [19], or may be single p53-mutant cells that colonize the uterine epithelium. Indeed, we have seen single cells emerging from the FTE and uterine luminal epithelium in a mouse model that generates both HGSC and USC [6]. As such, we believe lineage tracing studies in mammalian models may better ascertain the origin of USC development and its relationship to cell mobilization between the fallopian tube and uterus.

Previous studies are consistent with our intentionally narrowed analysis focused on limited origin sites. In the cell-of-origin TCGA Pan-Cancer Atlas study, the USC samples clustered in iCluster 23, which is comprised of HGSC (OV), lung cancers (LUAD and LUSC), and breast cancers (BRCA), alongside primarily USC but not type I endometrial cancers [73]. Since both USC and HGSC are nearly universal in p53 mutation, other studies have evaluated the p53 mutation within precursor-like lesions and the p53 mutation found in tumors and metastases. The data for these p53 mutations indicate a complex disease picture in which a handful of studies find the USC tumor p53 mutation in STIC-like or otherwise tubal lesions in the same patient (thereby consistent with the STIC/FTE-origin hypothesis) [18,74]. However, another study found USC p53 mutations were distinct, particularly with adnexal lesions [19]. There may be a more complex etiology; these were cohorts in different nations with different patient characteristics. The US-based study found p53 mutation conservation and may be consistent with the US patient population of increasing incidence. It will be of interest to deep-sequence mutations from co-incident STICs with USC cases, with or without metastases, to establish mutational phylogeny, as has been previously completed for HGSC.

## 5. Conclusions

There was a surprisingly high fraction of DNA methylation changes that were consistent between high-grade serous ovarian cancer, uterine serous carcinoma, and the fallopian tube epithelium, relative to normal uterus controls. This may explain clinical similarities in USC and HGSC phenotypes, and why USC is more deadly than Type I endometrial cancers. Since DNA methylation is often associated with development, these shared genes between USC and the FTE may be informative and supportive of the model that some USC tumors originate from the FTE and subsequently metastasize to the uterus. Gene hits will be useful in identifying novel treatment and diagnostic targets.

## Figures and Tables

**Figure 1:**
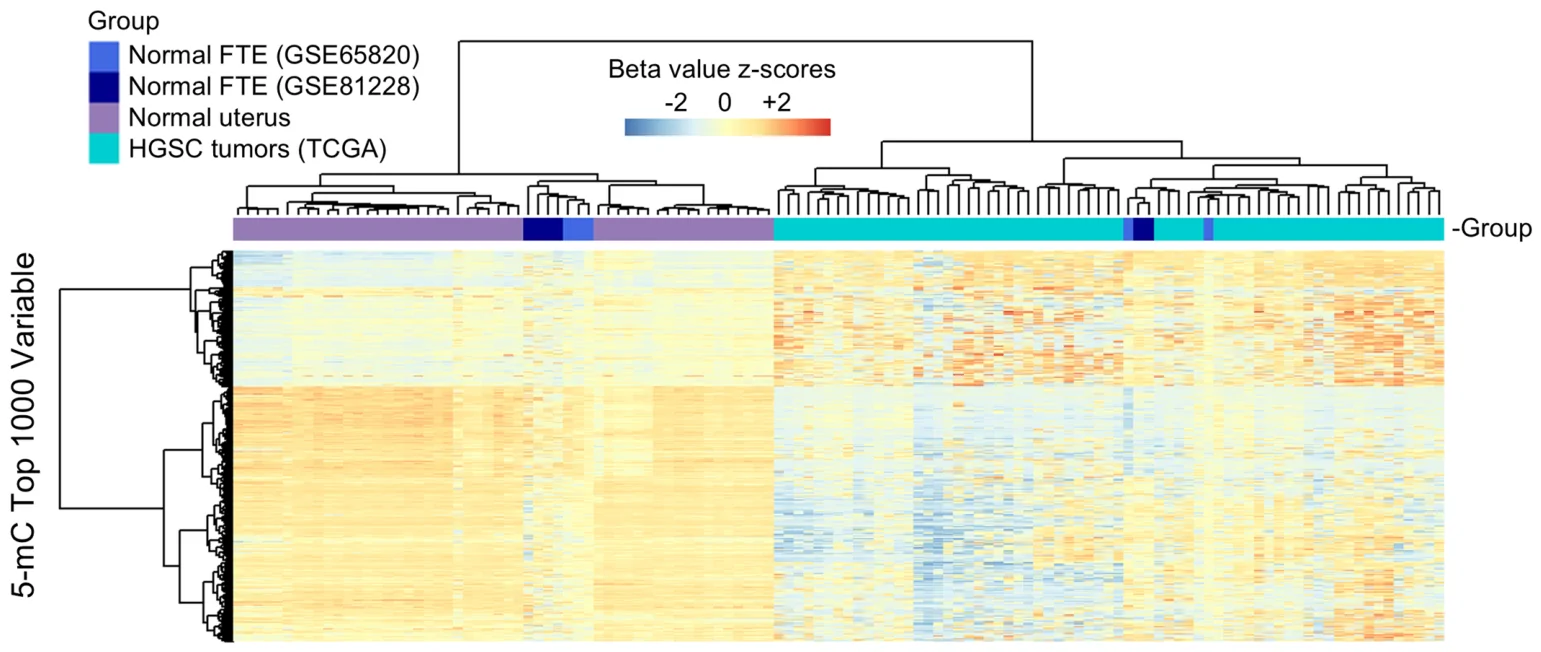
Hierarchical clustering of 5-mC DNA of HGSC with normal uterus and normal FTE. Row-scaled (each DNA methylation marker) beta values are shown. Ward.D2 clustering was used to produce hierarchy trees. A random selection of HGSC of similar size to the panel of normals is displayed for the top 1000 variable DNA methylation markers. Notably, different FTE datasets share similarities. HGSC clusters away from normal uterine patterns, and some FTE samples have HGSC-clustering patterning.

**Figure 2:**
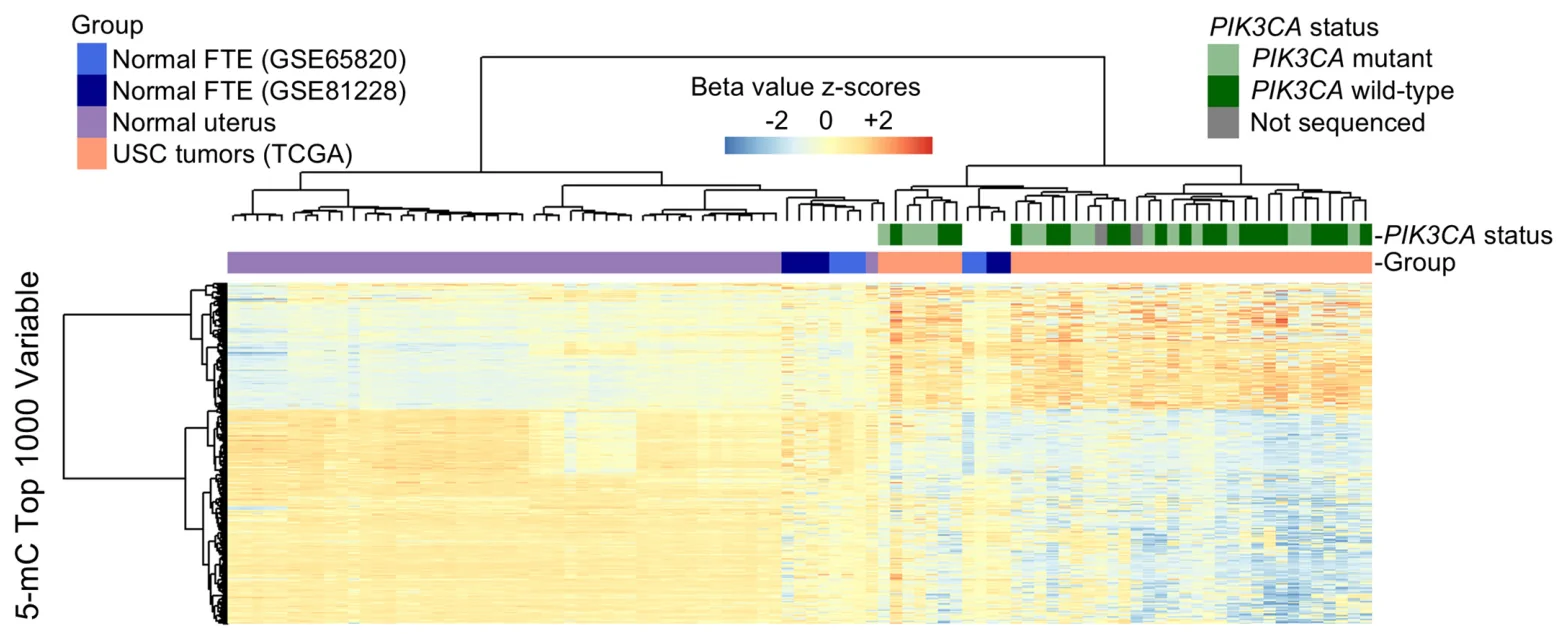
Hierarchical clustering of 5-mC DNA of USC with normal uterus and normal FTE. Row-scaled (each DNA methylation marker) beta values are shown. Ward.D2 clustering was used to produce hierarchy trees. USC tumors compared to the panel of normals are displayed for the top 1000 variable DNA methylation markers. Notably, different FTE datasets share similarities. USC clusters are distinct from normal uterine patterns, and some FTE samples have USC-clustering patterning.

**Figure 3:**
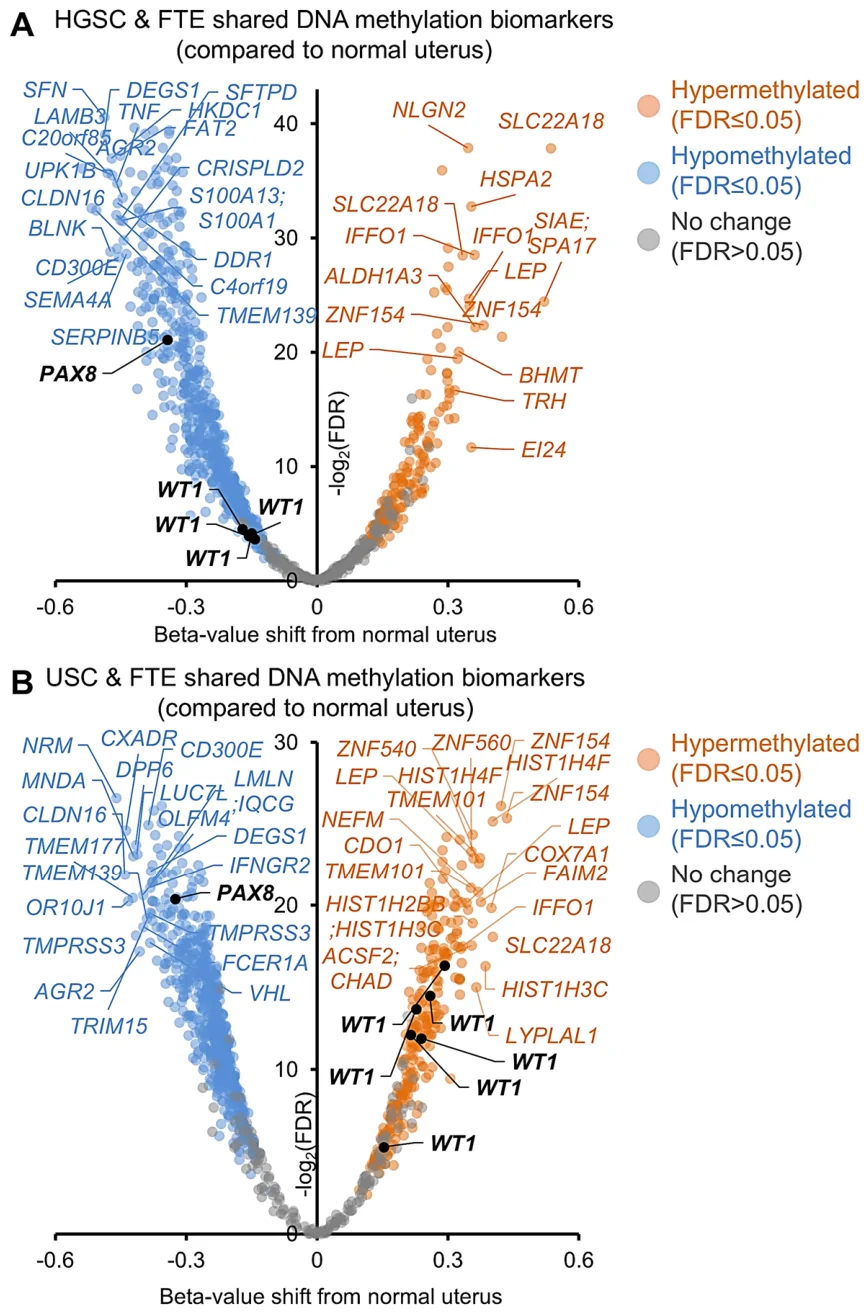
Gene-associated differential DNA methylation. (**A**) Markers from top 1000 variable in HGSC and FTE relative to uterus are plotted as hypermethylation (orange) or hypomethylation (blue). Mean differences in beta values between tumor and FTE samples compared to normal uterus samples are plotted. Top 20 genes by beta value are highlighted. *PAX8* and *WT1* are significantly hypomethylated and shown for reference. Multiple genes may be displayed with multiple DNA methylation events associated with the same gene. (**B**) Similar to (**A**), with significance based on both FTE and USC relative to normal uterus. Note the opposite *WT1* result, hypermethylation, in USC.

**Figure 4:**
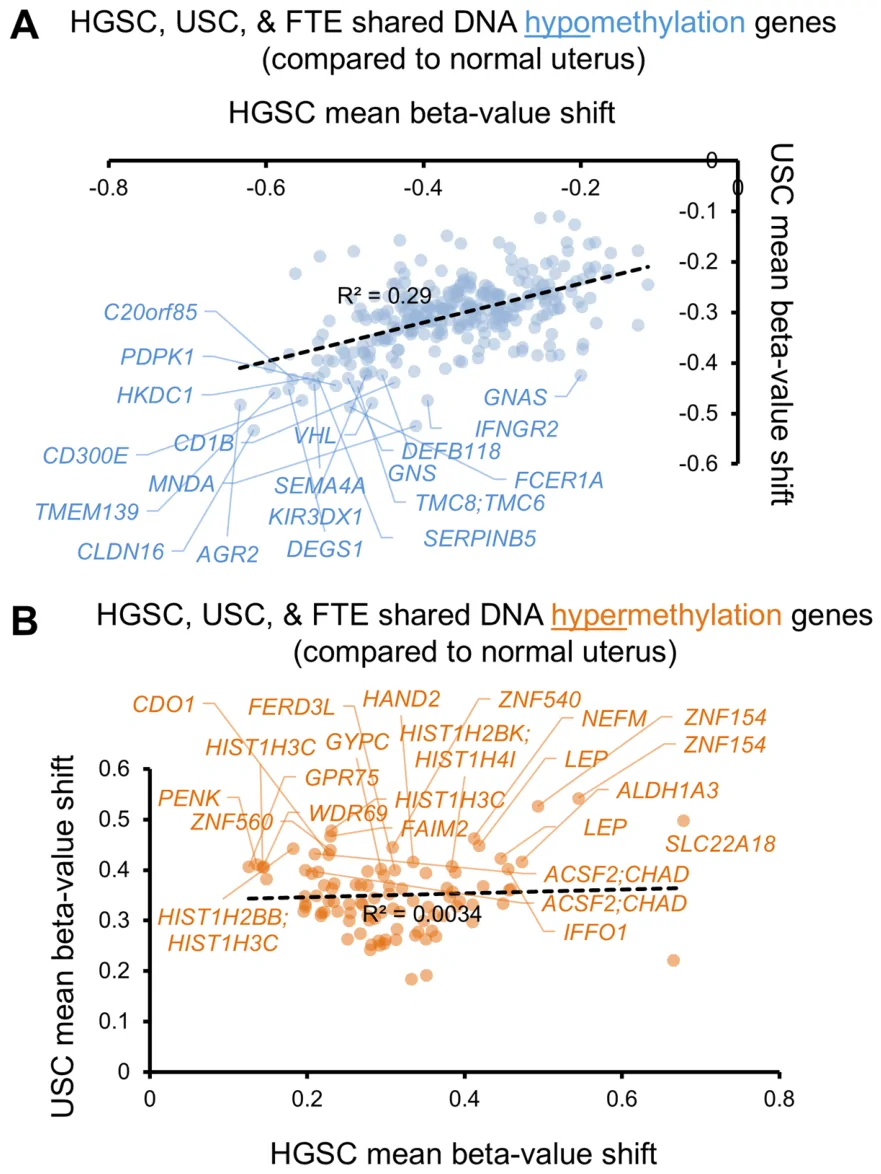
Correlation of DNA methylation with HGSC and USC shared biomarker genes. Top 20 differentially methylated non-redundant genes in the USC cohort are labelled. Shift in axes refers to the mean difference in beta values of tumor and FTE samples, compared to normal uterus samples. (**A**) Hypomethylated genes, with notable correlation. (**B**) Hypermethylated genes, without notable correlation.

**Figure 5:**
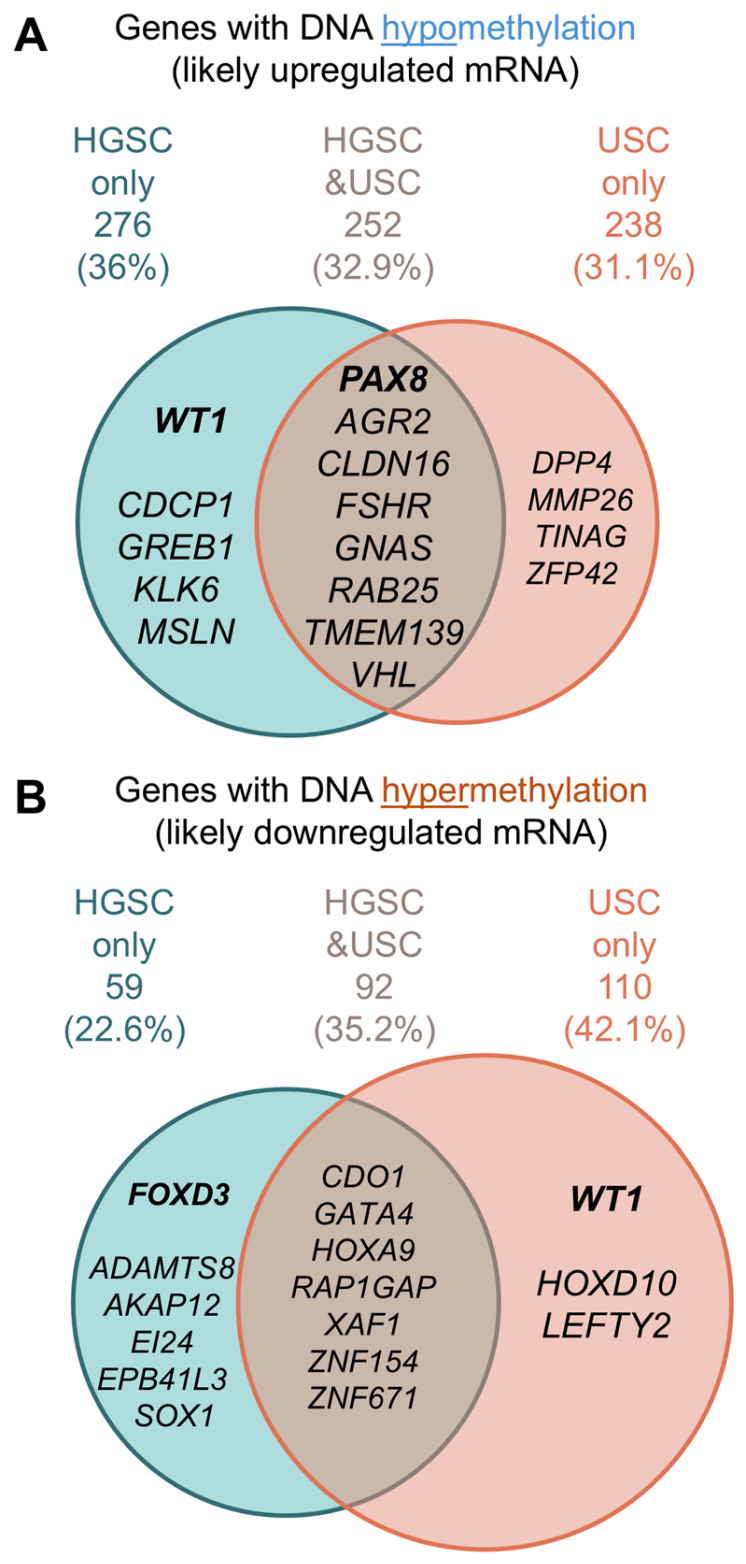
Venn diagram of shared and distinct differentially DNA methylated genes compared to normal uterus in HGSC and USC. Genes consistent or distinct within the top 1000 variable DNA methylation regions were analyzed for overlap between datasets. In all cases, genes were significant in cancer type labeled (HGSC or USC) and the FTE, relative to normal uterus. Genes noted in main text results are placed within their respective group for (**A**) hypomethylated genes and (**B**) hypermethylated genes.

## Data Availability

Data are publicly available under the accession numbers stated in the Materials and Methods Section.

## Supplementary Materials

The following supporting information can be downloaded at https://www.mdpi.com/article/10.3390/genes17101160/s1, Figure S1: Clustering results on control analyses; Figure S2: HGSC and USC combined hierarchical clustering; Table S1: USC and FTE variably DNA methylated genes compared to normal uterus; Table S2: HGSC and FTE variably DNA methylated genes compared to normal uterus; Table S3: Venn data of differential and shared DNA methylated genes.

## Abbreviations

The following abbreviations are used in this manuscript:

5-mC	5-methylcytosine
BSO	Bilateral salpingo-oophorectomy
CG	Cytosine–guanine
CUP	Cancer of unknown primary
DNMT	DNA methyltransferase
EMT	Epithelial-to-mesenchymal transition
FDR	False discovery rate
FIGO	International Federation of Gynecology and Obstetrics
FTE	Fallopian tube epithelium
GDC	Genomic Data Commons
GEMM	Genetically engineered mouse model
GEO	Gene Expression Omnibus
HGSC	High-grade serous ovarian carcinoma
IHC	Immunohistochemistry
OV	Ovarian cancer (TCGA designation)
PCA	Principal component analysis
SEIC	Serous endometrial intraepithelial carcinoma
STIC	Serous tubal intraepithelial carcinoma
TCGA	The Cancer Genome Atlas
UCEC	Uterine corpus endometrial carcinoma (TCGA designation)
USC	Uterine serous carcinoma
WT1	Wilms tumor 1
