# Supplementary material for: DNA Methylation Patterns Between Serous Uterine and Ovarian Carcinomas Indicate Shared Markers with Fallopian Tube Epithelium and Distinctions Consistent with Histopathology
Source: Genes (Basel). 2026 Sep 22;17(10):1160. doi: 10.3390/genes17101160 (PMC13704139; doi:10.3390/genes17101160)
Supplement: Supplementary file 1 [file genes-17-01160-s001.zip › SupplementalFigures.pdf]

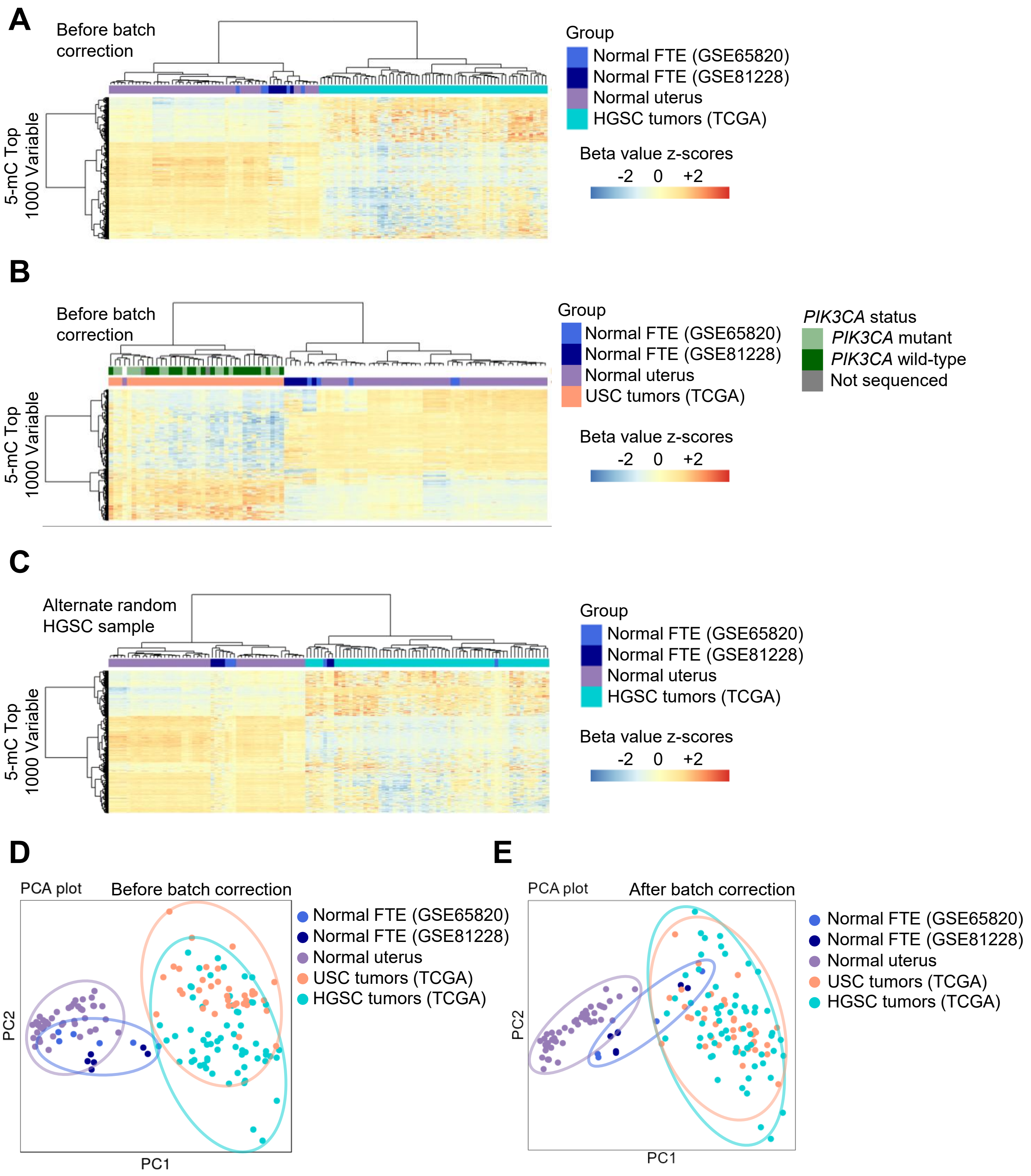

Supplementary Figure S1. Clustering results on control analyses. (A) Hierarchical clustering of HGSC before Combat batch effect correction. **(B)** Hierarchical clustering of USC before Combat batch effect correction. **(C)** Hierarchical clustering of HGSC, with batch correction, using an independent randomly sampled tumor cohort. **(D)** PCA plots of shared probe data (N= 487 probes) from Figures 1&2 before ComBat batch effect correction or **(E)** after batch effect correction. Ellipses of biological groups were manually added for visual reference and do not represent statistical assessments.

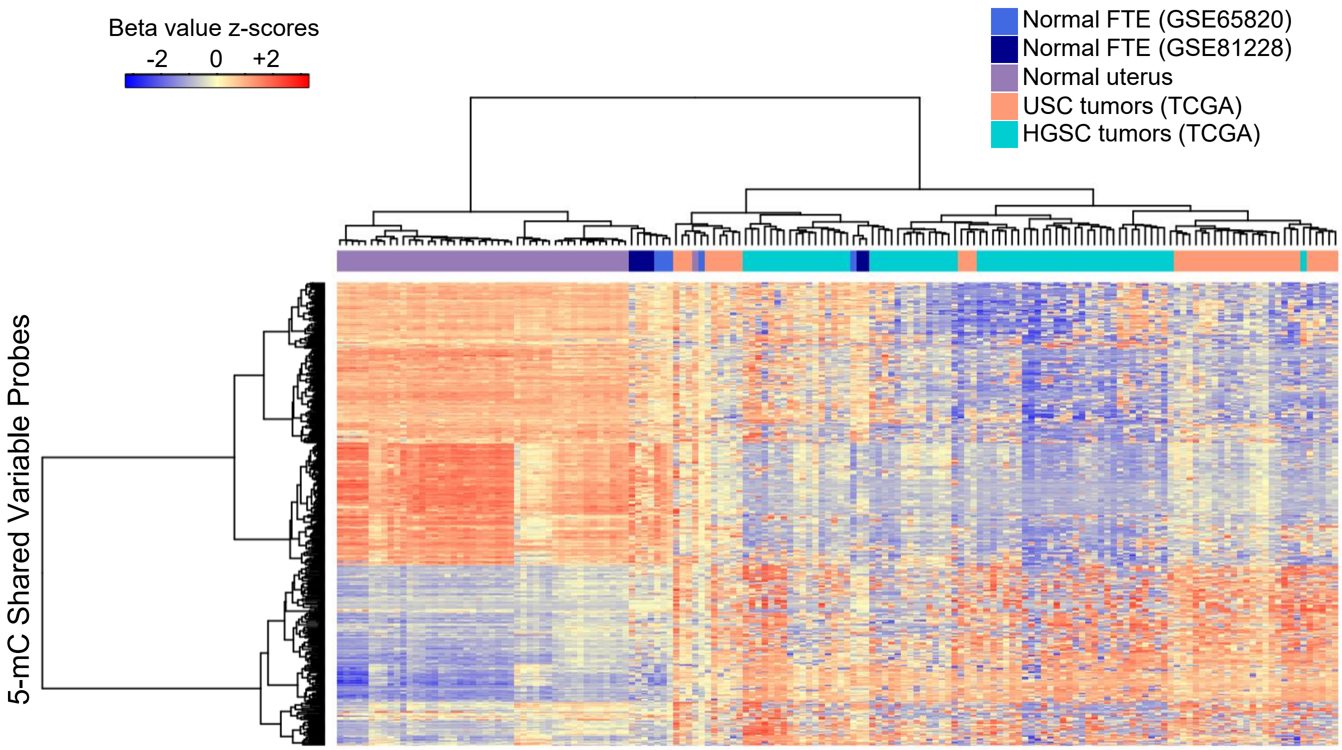

Supplementary Figure S2. HGSC and USC combined hierarchical clustering. Row-scaled (each DNA methylation marker) beta values z-scores are shown. Data from probes shared in Figures 1 and 2 were used (N= 487 probes). Ward.D2 clustering was used to produce hierarchy trees.
